# Case Report: Psychotherapy of a 10-year-old Afghani refugee with post-traumatic stress disorder and dissociative absences

**DOI:** 10.3389/fpsyt.2022.940862

**Published:** 2022-07-22

**Authors:** Nastia Junod, Olga Sidiropoulou, Daniel S. Schechter

**Affiliations:** ^1^Child and Adolescent Psychiatry Service, Lausanne University Hospital, Lausanne, Switzerland; ^2^Department of Psychiatry, Lausanne University Faculty of Biology and Medicine, Lausanne, Switzerland; ^3^Department of Child and Adolescent Psychiatry, New York University Grossman School of Medicine, New York, NY, United States

**Keywords:** post-traumatic stress disorder (PTSD), dissociation, refugee mental health, psychotherapy, post-traumatic play, intersubjectivity

## Abstract

Violence-related post-traumatic stress disorder (PTSD) in the context of war and terrorism has become an increasingly pressing public health issue relevant to refugee children and families. PTSD and related psychopathology in children can adversely affect all domains of development and, in particular, interfere with learning and socialization. When the experience of violent trauma and related loss is shared with the entire family, resulting impairment and distress may prevent caregivers from being psychologically available to process their traumatized children’s emotional communication and otherwise meet their children’s developmental needs. When children suffer from PTSD, it may be impossible to put their experience and related thoughts and feelings into words, let alone a coherent narrative. The latter difficulty can be even more pronounced when the child displays dissociative symptoms, possibly signaling a dissociative subtype of PTSD. Thus, the narrative within the child’s play during psychotherapy becomes all the more important as an indicator of the child’s internal world. This case report is an example both of evaluation and of psychotherapy that is both psychodynamic and trauma-informed with a 10-year-old Afghani boy who suffered the violent loss of his father at age of 3 years, leading to his immigration to Switzerland. This paper addresses the question of how the psychotherapist can accompany the child through the elaboration of his trauma and how the therapist can contribute to the co-construction of a coherent narrative of the child’s experience and to the restoration of an intersubjective connection between the traumatized child and caregiver.

## Introduction

Children are exposed to war, terrorism, and other forms of interpersonal violence worldwide. Psychological and developmental sequelae can be particularly noxious when any form of violence leads to parental loss. Post-traumatic stress disorder (PTSD) affects all domains of children’s social-emotional development, resulting often in disruptive behavior, heightened vigilance, and dissociative symptoms that ensue to protect the child from overwhelming threat, yet when generalized after the threat has passed, can interfere with learning and socialization. When multiple family members suffer from violence-related PTSD, their respective symptoms may prevent them from responding sensitively to each other’s suffering and developmental needs (1). It may be impossible for traumatized children to communicate their experiences, depending on their capacity for symbolization and verbal expression. Even if verbal, if the child has PTSD and, in particular, manifests a dissociative subtype of PTSD with prominent emotional numbing, derealization, and depersonalization (2), a coherent narrative about traumatic experiences may be extremely challenging. Play, especially for young children, becomes the primary means of communication. The more helpless and alone children feel in a violent environment, the more they try to control traumatic memories and related emotional dysregulation beyond their control through solitary play repeatedly reenacting the events (a.k.a. “post-traumatic play”) (3). One often neglected “side-effect” of post-traumatic play, is that it can trigger PTSD symptoms in the surviving family members, impairing the surviving parent’s emotional availability and ability to connect intersubjectively with their child (4).

In a trauma-informed psychodynamic psychotherapy model, the psychotherapist plays the role of a “mentalizing third” (5) who is an individual observer and not being part of the relational system of the two individuals coming for consultation—such as a mother-child dyad. This mentalizing third expressly thinks about and holds in mind the parent’s and child’s thoughts, feelings, and intentions, while also taking their perspective and potential biases into account. So, how can a psychotherapist—within that specific model of psychotherapy—endorse the function of that mentalizing third so that the parent and child can internalize or integrate that kind of metacognitive monitoring into their exchanges about their shared traumatic experiences—such as sudden loss—even in the absence of the psychotherapist?

## Case description

In this article, we will present a case report of the therapy that is both psychodynamic and trauma-informed, of Mustafa^1^ a 10-year-old Afghani immigrant to Switzerland referred for psychotherapy in the context of being assigned to a therapeutic school because of scholastic and behavioral problems. His teachers wondered whether he suffered from Attention-Deficit/Hyperactivity disorder (ADHD). During his first session, his therapist noted a lot of anxiety, which appeared to trigger his externalizing symptoms. Mustafa displayed multiple fears of injury and aggression triggered by ordinary loud sounds (e.g., pencils rolled in their aluminum box) or by peers (e.g., anger outbursts), and when asked about his mental “absences” Mustafa said—believing he was asleep—*“I had a nightmare.”* This curious symptom observed both in the classroom, in therapy sessions, and at home by his family contained elements of derealization (seems like a “nightmare”) and depersonalization (Mustafa saw himself “sleeping” as if being outside himself and sleeping at the same time). Further history revealed that Mustafa who is of Hazara ethnicity was forced to leave Afghanistan with his family in 2015 at 4 years old due to persecution of the Hazara and the murder of his father by the Taliban. He was shot at close range as he attempted to protect Mustafa’s older brother. Mustafa—who did not likely witness that—was in earshot, with his aunt rushing him to safety next door. After the murder, Mustafa, his mother, older brother, and sister left Afghanistan and arrived in Switzerland after a 3-month journey. His sister described their immigration as long, difficult, and triggering persistent fears of loss. It was fraught with the uncertainty of difficult passages in secret across mountainous regions in which there was a risk of further attacks within Afghanistan. They were unsure of whether they would have enough food and water, or if they would ever be able to return for their belongings as they were only able to take what they could carry in a small backpack. They did not know if they would ever again see the family whom they left behind. Subsequently, the family faced placement and resettlement in settlement camps where they feared illness, maltreatment, and met the uncertainty of not knowing where they would be sent next, if they would be able to stay together, and if they would be able to remain where they were sent and to thrive. Moreover, when the family finally arrived in Switzerland, they were left on their own, with very little information given about where they were, where they could get food, where they will be next and how long it would take to complete the immigration legal process.

## Diagnostic assessment

During his first school year in Switzerland, the patient presented with motor restlessness, difficulty concentrating and impulsive behavior, emotional dysregulation, as well as recurrent disruptive behavior in class including aggression toward his peers. For that reason, he was transferred to a therapeutic school (with fewer students and more teaching staff). There, the patient presented additional, transient “absences” during which the patient did not seem to hear the adults speaking to him and appeared to stare and to be internally preoccupied. He also displayed startle responses to abrupt sounds or agitation by his peers. After being startled, he seemed disorganized. At that time, he admitted having intrusive, frightening images of faceless men fighting, holding weapons, women and children crying, and nightmares of intruders, monsters. The patient also exhibited avoidance and emotional numbing during verbal and physical conflicts between classmates as well as hypervigilance and irritability. He complained of fatigue due to awakening in the night, worries about his mother and other people in Afghanistan, and fears that something bad would happen again to him and his family. Re-experiencing, avoidance, hyperarousal, and negative cognition symptoms according to the DSM-5—strongly suggested a diagnosis of PTSD related to early experiences in civil war-torn Afghanistan. Moreover, the patient’s symptoms involving the moments of absences—not consistent with any neurological difficulties, but rather feeling as if he was in a dream or movie and looking at himself and his classmates from afar (i.e., derealization and depersonalization), pointed toward the specific subtype of PTSD—called dissociative PTSD—which is characterized by abnormally high activation of several brain regions associated with emotional regulation and arousal modulation (2). We did not feel that Mustafa’s dissociative symptoms were pervasive enough to say that he had a dissociative disorder independent of PTSD of a dissociative subtype.

The patient’s clinical picture at the time could have also suggested complicated bereavement. However, given that Mustafa was only 3-year-old at the time of his father’s murder by the Taliban, he would not yet have been developmentally able to comprehend cognitively the finality of death—a cognitive milestone that only occurs around the average age of 7 years. Another differential diagnosis came to the professional’s mind: ADHD. Indeed, aside from the agitation and poor focus capacities, the patient was particularly absent during the play in therapy. Nevertheless, this can be understood by the fact that the symbolic play done during the psychotherapy sessions involved re-experiencing the traumatic events surrounding his father’s murder. The patient would present dissociative symptoms as mentioned above that can easily be confused with an attentional disorder. At the beginning of the therapy, we put this diagnosis of ADHD on hold as a pending treatment of the patient’s PTSD, knowing that the two disorders may be comorbid. However, in our opinion, the PTSD symptoms were more prominent, distressing, and troublesome at the time of assessment. However, after 2 years of therapy, while the PTSD symptoms were improving (fewer dissociative episodes, less conflict with peers, and hypervigilance), the patient was still having difficulty concentrating in the class as well as learning disabilities. Therefore, we decided to screen him for ADHD, which confirmed this co-morbid diagnosis.

## Course of psychotherapy

During the first 4-month-long phase of treatment, Mustafa was agitated, inattentive, and hypervigilant (e.g., abruptly looking at the door if he heard someone in the hall). He presented dissociative symptoms, such as those described above and also in the form of what could have been considered a hallucination if Mustafa had not been able to test reality just after the odd perception: while looking at a toy he said, *“I thought it was a severed head* [but I knew it was not].” Although he remained compliant, Mustafa’s play was disorganized, without significant interactions between characters (1). One character emerged in every session: an all-powerful monster—represented by a vampire—who repeatedly killed a “man,” a “sister,” or a “brother.” The killings that Mustafa staged were brutal, without escape, and the therapist felt like a helpless onlooker as Mustafa—with his back turned toward her—ignored and refused any help or participation in the play. During that phase, the mother was reluctant to see her son’s new therapist. Indeed, during the first interview at her son’s new school, she was asked to tell her story. Following this interview, Mustafa’s mother stated that she *“did not want to answer any more questions about Afghanistan.”*

During the second phase of psychotherapy, lasting 6 months, Mustafa repeated battle-scenes between a little boy, a sister, and a brother trying to escape a monster that came to harm, abduct, or kill them. As the therapeutic alliance strengthened, Mustafa allowed the therapist to join in his play and to insert a trustworthy policeman who restored justice. The combat and killing in his play decreased in intensity and frequency; the monster became less powerful or at least, easier to capture. The father’s character remained absent from the play: *“He’s dead”* or *“There is no father,”* declared Mustafa when his therapist asked him about the presence of his father. This shows how avoidant Mustafa had been concerning his father’s memory. His behavior mirrored the avoidance he experienced at home. His sister told the therapist that there were no pictures or mention of their father in the house. In addition, when Mustafa asked the therapist if she thought that he had *“the same hair as my father,”* she responded by asking him if his mother would know. Mustafa responded: *“Mom says that she cannot tell how he* [his father] *looked,” “I think he looked like nothing.”* Mustafa was thus very afraid to activate his mother’s PTSD symptoms. We speculate that Mustafa would not thereafter speak about his father or play out scenarios that he did during therapy, at home. During that time, the mother came for an interview. The therapist—seeing that she was very defensive and gaze-avoidant—said that *“she does not have to repeat her story.”* She encouraged the mother to talk about her son to create a safe place and positive alliance before eliciting the trauma narrative.

It would not be until after the summer, during the 2nd year and third phase of psychotherapy, that Mustafa spontaneously talked about his father. One day, he brought a drawing to his session—a school assignment to tell the story of a family member. Mustafa said, *“It’s for my father, but he cannot receive it because he is dead.”* Then, while mimicking pointing the gun at his head, he said, *“He (my father) was shot by the Taliban in the dark.”* Mustafa asked his therapist: *“Is your father dead too?”* to which she responded: *“No, but I would be very sad and angry if he was.”* Psychotherapy thus offered Mustafa a safe place with an emotionally present therapist with whom he could experience the existence of his father.

Since then, Mustafa’s play changed: a father-figure appeared and fought the monster with the boy. Turning passive to active, Mustafa could avert tragedy in play that he could not in life. The monster weakened and failed to kill more people. Subsequently transformed as a “teddy-bear-monster.” Mustafa described him as *“the little boy’s new friend,” “a part of the family.”* Fewer dissociative episodes were reported along with less agitation at school.

After 18 months into therapy, the United States Army exited Afghanistan, leaving it in the hands of the Taliban. Innocent people were hurt, killed, and forced to leave. This was extremely stressful for Mustafa and his family—because of their relatives and friends there. It triggered renewed helplessness in Mustafa’s post-traumatic play: the monster returned ferociously, and the father became a weaker character. The boy became “the hero of the story” who could save everybody. Mustafa described the mother as *“going crazy because she’s too stressed.”* She was left aside, unable to fight the monster *“because she’s too tired.”* Mustafa presented characters who were dismembered, echoing news that a cousin’s hands were “chopped off” by the Taliban. At that time, the therapist saw the mother again. She appeared depressed, fearful for her brother in Afghanistan, and described his condition there: *“He has to hide from the Taliban, they come into people’s home and kill and mutilate them.”* Nevertheless, Mustafa’s mother was less defensive than previously. The therapist was able to tell her that her son *“had lived really difficult things and that he is traumatized and that he needs therapy to speak about that trauma.”* She listened tearfully. The mother then said of Mustafa playing at home: *“He makes up stories in French, which I cannot understand.”* When the therapist asked her if Mustafa was speaking about Afghanistan at home, she replied that her son *“never speaks about Afghanistan at home*… *maybe he thinks that it can make me sad.”* At that moment, the therapist, seeing that the alliance was positive and that the mother would speak about her and her son’s trauma, said to the mother: *“You are both in your own pain and I think it will be less frightening if you come together. My role is to accompany you both in that.”* To which the mother replied: *“Ok, when can I see you again?”*

## Timeline


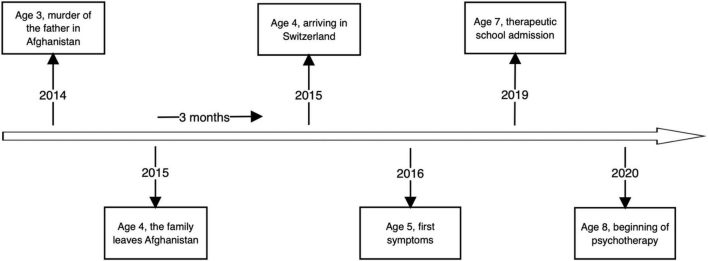


## Discussion

This report presented the case of a 10-year-old Afghani refugee boy who at the age of 3 years, experienced the murder of his father in the context of civil war, and then the stress of immigration and loss of his homeland. Post-traumatic play therapy following these experiences, that is to say, both psychodynamic and trauma-informed allows for imaginal exposure to traumatic memory traces, memory recontextualization, and reconsolidation with help of the therapist’s mentalizing stance (mentalizing third). In this psychotherapy, the therapist helped the patient clarify the Taliban’s murder of his father and loss of homeland that Mustafa had experienced as a helpless preschool-age child. Mustafa could, during the course of psychotherapy at the age of 10 years, form a more coherent trauma narrative, rediscover earlier memories of shared experiences while his father was still alive, and also confront and attribute meaning to the pain of his and his mother’s losses, she having also likely suffered from complicated bereavement. Mustafa’s empowerment through the restoration of justice in his play with the help of his therapist allowed what had been frozen in the compulsive repetition of post-traumatic play to evolve, while simultaneously distinguishing Mustafa’s safe, present context in Switzerland from his dangerous, past context in Afghanistan. The connection between the child and the therapist permitted the sharing of an intersubjective field that had been foreclosed in his relationship with his traumatized mother who could not intersubjectively join with him despite their shared traumatic experiences because of her own complicated bereavement, PTSD symptoms, and depressive state that ensued. The authors assert that mutative effects were enhanced in the treatment by the psychotherapist who played the role of a mentalizing, emotionally regulating third (5), who helped explicitly to link memory traces, affects, and behaviors related to Mustafa’s traumatic experiences that had been dissociated from one another. During the course of this 2-year long psychotherapy, the patient and the family’s avoidance of traumatic and non-traumatic memories of the lost father diminished and the patient’s PTSD and dissociative symptoms remitted, and his functioning improved both scholastically, socially, and with respect to his relationship with his deeply traumatized mother.

## Patient perspective

At the beginning of the psychotherapy—as described above—it was difficult if not impossible for the therapist to talk about the trauma with the patient. Gradually, the therapist was able to bounce back and forth on the elements brought into the trauma play by the patient and name the difficult events the patient had experienced. For example: *“This little boy in the story looks very scared. Have you ever been very scared like the little boy?”* To which the patient would respond, *“Yes, for example in Afghanistan.”* Following these brief inclusions during the sessions, the therapist was able to name the patient’s trauma: *“You experienced difficult events when you were a child, in Afghanistan, and here you can talk about them so that they do not come back to you all of a sudden, during the day, at times when you do not want them to come back and where they can scare you.”* The trauma named clearly in the therapy, gave the patient the possibility to talk about it spontaneously in the game or by revealing his worries: *“Are there Taliban in Switzerland?”*

Similar work could be done with the patient’s sister and mother. During the first few meetings, it was impossible to talk about the trauma. Mustafa’s mother refused to talk about Afghanistan and the trip to Switzerland, and the sister denied that the patient could remember anything at all, given his young age. As the therapeutic alliance strengthened, the therapist was able to reveal elements of the sessions to his family: *“He makes up a lot of stories about people fighting, about children using guns, and he tells me that he remembers that in Afghanistan young children have guns. He is also very afraid of loud noises* [that sound like gunfire]. *Does this sound familiar?”* To which the family responds, “*In Afghanistan there are sounds of shooting and bombs often. Maybe he remembers that.”* From that point on, it was easier for the family to accept the patient’s psychotherapy because they could understand its importance in making sense of Mustafa’s early experience of the trauma and loss of war and subsequent displacement.

## Data availability statement

The datasets for this article are not publicly available due to concerns regarding participant/patient anonymity. Requests to access the datasets should be directed to the corresponding author.

## Ethics statement

Ethics review and approval/written informed consent was not required as per local legislation and institutional requirements.

## Author contributions

OS: scientific contribution. All authors contributed to the article and approved the submitted version.

## Conflict of interest

The authors declare that the research was conducted in the absence of any commercial or financial relationships that could be construed as a potential conflict of interest.

## Publisher’s note

All claims expressed in this article are solely those of the authors and do not necessarily represent those of their affiliated organizations, or those of the publisher, the editors and the reviewers. Any product that may be evaluated in this article, or claim that may be made by its manufacturer, is not guaranteed or endorsed by the publisher.

## Footnotes

1 Fictitious name.
