# Erratum: Case Report: Psychotherapy of a 10-year-old Afghani refugee with post-traumatic stress disorder and dissociative absences

**DOI:** 10.3389/fpsyt.2023.1182111

**Published:** 2023-03-16

**Authors:** Frontiers Production Office

**Affiliations:** Frontiers Media SA, Lausanne, Switzerland

**Keywords:** post-traumatic stress disorder (PTSD), dissociation, refugee mental health, psychotherapy, post-traumatic play, intersubjectivity

An omission to the funding section of the original article was made in error. The following sentence has been added: “Open access funding was provided by the University of Lausanne.”

The original article has been updated.

